# Supplementary material for: The lipid transfer protein Saposin B does not directly bind CD1d for lipid antigen loading
Source: Wellcome Open Res. 2019 Oct 18;4:117. Originally published 2019 Aug 2. [Version 2] doi: 10.12688/wellcomeopenres.15368.2 (PMC6807164; doi:10.12688/wellcomeopenres.15368.2)
Supplement: Peer review history

# Peer review: Reviewer response for version 2

**DOI:** 10.21956/wellcomeopenres.16966.r36814

**Published:** 2019-10-25

**Authors:** Mariolina Salio

**Affiliations:** 1 Radcliffe Department of Medicine, Medical Research Council human Immunology Unit, Medical Research Council Weatherall Institute of Molecular Medicine, University of Oxford, Oxford, UK

I am satisfied with the revised version and the response to my queries.

I confirm that I have read this submission and believe that I have an appropriate level of expertise to confirm that it is of an acceptable scientific standard.

# Peer review: Reviewer response for version 2

**DOI:** 10.21956/wellcomeopenres.16966.r36816

**Published:** 2019-10-21

**Authors:** Tim P. Levine

**Affiliations:** 1 Institute of Ophthalmology, University College London, London, UK

All required changes made, as described in the Authors' Rebuttal.

I confirm that I have read this submission and believe that I have an appropriate level of expertise to confirm that it is of an acceptable scientific standard.

# Peer review: Reviewer response for version 2

**DOI:** 10.21956/wellcomeopenres.16966.r36815

**Published:** 2019-10-21

**Authors:** Weiming Yuan

**Affiliations:** 1 Department of Molecular Microbiology and Immunology, Keck School of Medicine, University of Southern California, Los Angeles, CA, 90033, USA

The authors have addressed my concerns and comments.

I confirm that I have read this submission and believe that I have an appropriate level of expertise to confirm that it is of an acceptable scientific standard.

# Peer review: Reviewer response for version 1

**DOI:** 10.21956/wellcomeopenres.16790.r36454

**Published:** 2019-09-30

**Authors:** Mariolina Salio

**Affiliations:** 1 Radcliffe Department of Medicine, Medical Research Council human Immunology Unit, Medical Research Council Weatherall Institute of Molecular Medicine, University of Oxford, Oxford, UK

Shamin and colleagues investigate whether the LTP Saposin B directly binds to CD1d molecules for lipid antigen loading. It is known from the literature - well quoted in the manuscript - that Saposin B is the main regulator of CD1d-lipid antigen presentation to iNKT cells but to date it is not clear whether the two proteins directly interact.

 Using highly purified proteins, glycosylayed and not, and through different biochemical methods (in vitro equilibrium binding assay, in vitro cross linking and crystallization trials) the authors conclude there is no direct binding between CD1d and Saposin B.

 The group led by JE Deane has expertise in the field of GSL metabolism, and has used similar methods to prove interactions of Saposin A with GALC, including careful evaluation of the role of pH, presence or absence of lipids, influence of NaCl on electrostatic interactions. To this regard, however, it is not clear if the authors have tried the equilibrium binding assay of unloaded CD1d-b2m with aGalCer loaded Saposin B as additional control in Figure 2. This could be an important discriminator in the ability of the two proteins to interact.

 Although a negative result is always difficult to interpret (and publish), the authors discuss the limitations of their in vitro results, as well as that of previous assumptions stemming from knock out models where the lysosomal lipidome is severely altered. Overall, the results are in agreement with the fundamental distinct mode of action of Saposin C (which has been shown to interact with CD1b and CD1c) and Saposin B. Future studies looking at the ligandome of CD1d associated proteins in the lysosome may shed light on perhaps a larger loading complex which includes Saposin B.

I confirm that I have read this submission and believe that I have an appropriate level of expertise to confirm that it is of an acceptable scientific standard, however I have significant reservations, as outlined above.

# Response

**Published:** 2019-10-14

**Authors:** Maria Shamin

**Affiliations:** University of Cambridge, UK

Thank you for your thoughtful comments and constructive feedback. 

*It is not clear if the authors have tried the equilibrium binding assay of unloaded CD1d-b2m with aGalCer loaded Saposin B as additional control in Figure 2. This could be an important discriminator in the ability of the two proteins to interact.*

This important concern is similar to that raised by reviewer 1. We have addressed this concern by performing an additional equilibrium binding assay to test the binding of α-GalCer loaded SapB to CD1d loaded with endogenous lipids (Fig. 2D). Although this does not represent an “unloaded CD1d-β2m”, as mentioned in this comment, this experiment includes the endogenous lipid that co-purifies with CD1d following purification from human cells and therefore closely-resembles the state of CD1d that would be expected to occur in cells. As mentioned in response to reviewer 2, the relevance of an “empty” or “unloaded” CD1d for these assays is questionable as CD1d purified from insect or mammalian cells always appears to have lipids bound.

# Peer review: Reviewer response for version 1

**DOI:** 10.21956/wellcomeopenres.16790.r36344

**Published:** 2019-09-16

**Authors:** Tim P. Levine

**Affiliations:** 1 Institute of Ophthalmology, University College London, London, UK

This work sets out to show if saposins bind directly to CD1, which is relavant to the mechanism lying behind lipid loading into CD1, which is known to require saposins. Multiple biochemical techniques, some highly demanding, were performed with purified proteins and with great attention to detail and very clear controls.

 My major point about this work is that I could not tell which, if any, experiments also included relevant lipid, particularly pre-loaded into saposin. Either key sentences in the MS should be rewritten to avoid that impression (if it is wrong), or the MS should clearly include the caveat that some aspects of the physiological situation were not reproduced in the binding experiments. On that basis, it has not been addressed if prolonged interaction only occurs when one of the proteins is occupied by lipid. This might even be asymmetric so that prolonged interaction requires lipid in Saposin, but CD1 must be empty. 

 The rationale for this work is explained in 2 sentences of the final paragraph of the Introduction: "…several references in the literature [about] an inability to measure a direct interaction between saposins and human CD1d [3 refs]. Unfortunately, these reports do not show any data or detail the approaches used to monitor binding and the implication is that these interactions exist but are weak and very transitory.”

     This rationale is not explained as clearly as it might be. In the 1 ^st^ sentence, do the authors mean that the 3 refs were written to indicate that the lipid binding proteins must be able to bind each other even when lipid is absent to have a productive relationship for lipid handover? The different models should be described more, and the limits to the data mentioned in the 2 ^nd^ sentence should be outlined. In addition, the Introduction and Discussion should both describe (in brief and extensive terms, repsectively) what is known for other examples of lipid handover: NPC1/NPV2 MPT/ApoB LBP/CD14 CD14/MD2. Also in discussion of tapasin, describe if the relationship parallels the one envisaged here. Finally, is there handover of peptide from another binding protein to MHC?

 The estimated maximum level of Kd that these techniques would find is 40 μM (Fig 2B). The authors should discuss the physiological relevance of an interaction weaker than that. They should also discuss how proximity labelling (BioID, APEX) might find that. 

 State that SapB used might lack PTM required for binding in Fig 2, although this was tested in Fig 3. 

 State whether conditions in Fig 2C were shown to permit transfer of lipid. Ideally, this should be shown for a parallel experiment under the same conditions.

I confirm that I have read this submission and believe that I have an appropriate level of expertise to confirm that it is of an acceptable scientific standard, however I have significant reservations, as outlined above.

# Response

**Published:** 2019-10-14

**Authors:** Maria Shamin

**Affiliations:** University of Cambridge, UK

Thank you for your thoughtful comments and constructive feedback. In-line responses to your queries are included below. 

*My major point about this work is that I could not tell which, if any, experiments also included relevant lipid, particularly pre-loaded into saposin. Either key sentences in the MS should be rewritten to avoid that impression (if it is wrong), or the MS should clearly include the caveat that some aspects of the physiological situation were not reproduced in the binding experiments.*

Most experiments were carried out with SapB and CD1d-β2m loaded with the lipids they co-purified with following protein expression. Based on previous studies, these are likely to be *E.coli* phosphatidylethanolamine for SapB and endogenous human lipids that bind during folding in the endoplasmic reticulum and trafficking through the Golgi apparatus for CD1d-β2m (primarily sphingomyelin). As phosphatidylethanolamine is a lipid species that has been shown previously to bind CD1d then the proteins *as purified* are not necessarily in a non-physiologically relevant lipid-bound state. However, we accept that in light of no detectable interaction this is a crucial factor to be clear about in the text. To ensure that the lipids used in each experiment is clear, we have modified the relevant sections of the main text and figures (also see next comment).

*On that basis, it has not been addressed if prolonged interaction only occurs when one of the proteins is occupied by lipid. This might even be asymmetric so that prolonged interaction requires lipid in Saposin, but CD1 must be empty.*

We agree with the reviewer that the lipid-bound state of the protein components may be a crucial factor in the affinity and on/off-rates of their potential interaction. An experiment to try and address this, suggested by reviewer 1, is now included where the SapB has been exchanged to bind α-GalCer and the CD1d used with endogenous lipids, similar to those that would be bound upon delivery to the endocytic compartments where exchange should occur. This did not result in significant depletion of SapB in equilibrium binding assays (Fig. 2D) suggesting that if the lipid-bound state of these components is a driving factor in the interaction it is sufficiently complex to be outside the scope of this study. Regarding the reviewer’s statement that CD1d “must be empty”, it is unclear that CD1d actually ever exists in a truly empty form as it appears to always co-purify, from insect or mammalian cells, with lipids bound.

*The rationale for this work is explained in 2 sentences of the final paragraph of the Introduction. This rationale is not explained as clearly as it might be. In the 1st sentence, do the authors mean that the 3 refs were written to indicate that the lipid binding proteins must be able to bind each other even when lipid is absent to have a productive relationship for lipid handover?*

Statements in the referenced papers are not specific about what conditions the individual proteins might be in regarding lipid content for an interaction to occur. Despite this, all references agree that Saposins are crucial for lipid loading of CD1. In the review by Teyton 2018, he states “We have not been able to reliably measure an interaction between any mouse or human CD1 molecules and the four Saps…”. In both Yuan 2007 and Salio 2013 they reference previous work identifying interactions between CD1b and/or CD1c with Saposin C but both state that they have been unable to identify direct interactions of saposins with CD1d despite these papers being specifically focused on the role of CD1d and SapB in lipid loading. As the referenced papers are not specific enough in their statements to address the reviewers points we do not feel we can substantially re-write this statement. The lack of detail in the published statements were part of the motivation for our own studies and the range of techniques and conditions we tested.

*The different models should be described more, and the limits to the data mentioned in the 2nd sentence should be outlined.*

As above, we do not feel we can provide more detail as there were no models proposed and no data provided in the references. We have however, extended the first part of the last paragraph of the Introduction based on this reviewer’s next comment to try and clarify that a direct interaction was expected for this process.

*In addition, the Introduction and Discussion should both describe (in brief and extensive terms, repsectively) what is known for other examples of lipid handover: NPC1/NPV2 MPT/ApoB LBP/CD14 CD14/MD2.*

We thank the reviewer for highlighting these additional protein complexes involved in lipid handover and agree that this is an important addition as it helps put the current work in perspective regarding the requirement, in these other systems, for direct protein-protein interactions for lipid transfer. This is especially compelling as the affinities described for these interactions would have been clearly detectable in the assays used here. We have rewritten the final paragraph of the introduction to cite these examples of lipid handover that involve a direct interaction between proteins involved in lipid transfer. We have also added a substantial paragraph to the discussion where we discuss the mechanisms of lipid transfer between these proteins and the requirement for medium-to-strong, direct interactions between proteins engaged in lipid transfer.

*Also in discussion of tapasin, describe if the relationship parallels the one envisaged here. Finally, is there handover of peptide from another binding protein to MHC?*

The original comparison of saposin function to tapasin function was made by Salio *et al.* in their 2013 paper. The parallels envisaged between these systems are the requirement for a direct protein-protein interaction (MHC I to Tapasin versus CD1d to SapB) and the functional outcome of this interaction being a change to the antigen content of the binding groove (MHC I-peptide versus CD1d-lipid). Tapasin has been shown to bind directly to MHC I and functions to load and optimize the peptide binding to MHC I. There are clearly potential differences between these systems particularly as the relevant antigens have different solubility and thus peptides do not require “transfer proteins”. We have added a sentence to the discussion describing more clearly the potential parallels between these systems. However, we feel that any further speculation at this stage is unwarranted.

*The estimated maximum level of Kd that these techniques would find is 40 μM (Fig 2B). The authors should discuss the physiological relevance of an interaction weaker than that.*

We thank the reviewer for this point and how it relates also to the additional protein-protein interactions mentioned above involved in lipid transfer. The published affinities for these interactions range from 5 nM to 2 µM thus readily detectable using the techniques adopted in our study. An interaction weaker than 40 µM falls outside what would be considered a physiologically relevant interaction affinity in the absence of a highly-multivalent interaction, which we do not expect for SapB and CD1d. We have now added additional text to the discussion explicitly stating this point.

*They should also discuss how proximity labelling (BioID, APEX) might find that.*

Although BioID and APEX are excellent techniques for detecting transient and weak protein-protein interactions in cells, these techniques are not yet available for the analysis of interactions within the endolysosome due to the low pH of these compartments that significantly reduce the efficacy of the biotinylation reaction. We would be very excited to implement these sorts of techniques when they become available.

*State that SapB used might lack PTM required for binding in Fig 2, although this was tested in Fig 3.*

The reviewer is correct that the SapB used in Fig 2 is not glycosylated. However, previous *in vitro* lipid loading experiments (Salio 2013; Yuan 2007) did not use glycosylated SapB, indicating that this PTM is not necessary for function. Our experiment using glycosylated SapB in Fig. 3 was performed in the hope that this may increase the affinity to detectable levels, rather than be required for it. We have ensured this point is now clearer in the text.

*State whether conditions in Fig 2C were shown to permit transfer of lipid. Ideally, this should be shown for a parallel experiment under the same conditions.*

The conditions used in our binding experiments fall within the range of different conditions used in previous studies with SapB and CD1d. The pH and salt concentration chosen for this assay were based on previous studies, particularly Yuan 2007. For technical reasons we also included 0.05% digitonin. However, we note that the SPR experiment performed by Salio 2013 also included detergent, 0.005% surfactant P20.

# Peer review: Reviewer response for version 1

**DOI:** 10.21956/wellcomeopenres.16790.r36119

**Published:** 2019-08-29

**Authors:** Weiming Yuan

**Affiliations:** 1 Department of Molecular Microbiology and Immunology, Keck School of Medicine, University of Southern California, Los Angeles, CA, 90033, USA

Saposin B has been reported to mediate the lipid loading to CD1d in lysosomal compartments. This report employed several sensitive analytical methods to dissect whether saposin B directly interacts with CD1d/beta2m to deliver the lipid antigens to CD1d. The authors have extensively explored different experimental conditions to analyze the potential interactions with equilibrium binding assay, chemical cross-linking and co-crystallization methods. None of these assays detected direct saposin B-CD1d/beta2m interaction. The authors concluded that saposin B does not directly bind CD1d lipid loading.

 While the authors' studies are particularly thorough and extensive, the reviewer still has reservation to make a statement that this work could establish "comprehensively" that the role of saposin B in lipid loading does not involve direct interaction with CD1d. As the authors mentioned that the " *in vivo*" condition may be understandably different from the *in vitro* experimental conditions the authors have extensively tried. For example, as the authors pointed out, in lysosomes, there are multiple saposins present at possibly higher local concentrations, the potential "loading complex" may requires additional proteineous factors, etc. Therefore, while all results strongly suggested there might not be direct interaction, it is still hard to conclusively state there is none. 

 The additional factor is the pH in experimental condition may still affect the potential interaction even though it may not be critical for saposin and other lipid hydrolases.    

 Other comments:

1. The amount of cross-linked saposin/CD1d may be lower than detection limit in Commassie blue staing. The authors may consider using western blotting to increase the detection sensitivity.
2. In equilibrium binding assay with a-GalCer, the lipid exchange/editing can be imagined as unloading of "endogenous" lipids in CD1d (co-purified from bacteria or mammalian cells) and replacing with a-GalCer. It is unknown if both saposin and CD1d loaded with the same a-GalCer lipid, whether this editing and saposin/CD1d interaction would occur. As antigen editing would be stronger affinity antigen replacing the lower affinity antigen. It is strongly suggested that the same experiment in Fig. 2C to be repeated with purified CD1d (no a-GalCer) with a-GalCer-loaded saposin B.

I confirm that I have read this submission and believe that I have an appropriate level of expertise to confirm that it is of an acceptable scientific standard, however I have significant reservations, as outlined above.

# Response

**Published:** 2019-10-14

**Authors:** Maria Shamin

**Affiliations:** University of Cambridge, UK

Thank you for your thoughtful comments and constructive feedback. In-line responses to your queries are included below. 

*While the authors' studies are particularly thorough and extensive, the reviewer still has reservation to make a statement that this work could establish "comprehensively" that the role of saposin B in lipid loading does not involve direct interaction with CD1d. As the authors mentioned that the "in vivo" condition may be understandably different from the in vitro experimental conditions the authors have extensively tried. For example, as the authors pointed out, in lysosomes, there are multiple saposins present at possibly higher local concentrations, the potential "loading complex" may requires additional proteineous factors, etc. Therefore, while all results strongly suggested there might not be direct interaction, it is still hard to conclusively state there is none.*

We acknowledge that the lack of a detectable interaction does not necessarily mean that there is no interaction. We have therefore removed the word “comprehensively” from the abstract so that the conclusion now reads: “This work strongly indicates that the role of SapB in lipid loading does not involve direct binding to CD1d.”

*The additional factor is the pH in experimental condition may still affect the potential interaction even though it may not be critical for saposin and other lipid hydrolases.*

The equilibrium binding assays and cross-linking experiments were performed at pH 7. As described in the text, this is a pH previously demonstrated to be able to facilitate SapB loading of lipids onto CD1d. Although this may not represent the most optimal pH for the interaction it was a necessary compromise that supported both the technical requirements of our assays and a pH at which an interaction should have been observable.

*The amount of cross-linked saposin/CD1d may be lower than detection limit in Commassie blue staining. The authors may consider using western blotting to increase the detection sensitivity.*

Although we agree that Western blot would have the capacity to visualize weaker bands following cross-linking, there is one primary reason we have not used this approach here. If the interaction between SapB and CD1d is so weak that, even in the presence of high concentrations of purified proteins and crosslinker, the proportion forming a complex must be detected by Western blot then to us it seems likely this interaction is too weak for further structural analysis and unlikely to be of physiological relevance (in agreement with our equilibrium binding analysis).

*In equilibrium binding assay with a-GalCer, the lipid exchange/editing can be imagined as unloading of "endogenous" lipids in CD1d (co-purified from bacteria or mammalian cells) and replacing with a-GalCer. It is unknown if both saposin and CD1d loaded with the same a-GalCer lipid, whether this editing and saposin/CD1d interaction would occur. As antigen editing would be stronger affinity antigen replacing the lower affinity antigen. It is strongly suggested that the same experiment in Fig. 2C to be repeated with purified CD1d (no a-GalCer) with a-GalCer-loaded saposin B.*

We thank the reviewer for this suggestion and have now performed this additional experiment. We have repeated the equilibrium binding assay with SapB pre-loaded with α-GalCer, and CD1d loaded with the endogenous lipids with which it co-purifies following expression in a human cell line. In agreement with our other results, this alternate lipid loading of the protein components did not result in depletion of SapB from the supernatant even in the presence of 40 µM CD1d-β2m. As this additional experiment addresses an important point regarding relevant lipid loading (mentioned also by the other reviewers), we have updated the manuscript to include this new equilibrium binding assay in Fig.2, panel D.
